# Supplementary material for: End Sequence Analysis Toolkit (ESAT) expands the extractable information from single-cell RNA-seq data
Source: Genome Res. 2016 Oct;26(10):1397–410. doi: 10.1101/gr.207902.116 (PMC5052061; doi:10.1101/gr.207902.116)
Supplement: Supplemental Material [file supp_gr.207902.116_Supp_Materials.tar.gz › ESAT-esat_v0.1/doc/html/classbroad_1_1projection_1_1gsa_1_1_gene_set_enrichment.html]

ESAT: broad.projection.gsa.GeneSetEnrichment Class Reference


ESAT


- Main Page
- Related Pages
- Packages
- Classes
- Files

- Class List
- Class Index
- Class Hierarchy
- Class Members

All Classes Namespaces Files Functions Variables Enumerator Pages

- broad
- projection
- gsa
- GeneSetEnrichment

Public Member Functions |
List of all members

broad.projection.gsa.GeneSetEnrichment Class Reference

Collaboration diagram for broad.projection.gsa.GeneSetEnrichment:

[legend]

|  |  |
| --- | --- |
| Public Member Functions | |
|  | GeneSetEnrichment (MatrixWithHeaders data, Collection< String > group1, Collection< String > group2, Map< String, Collection< String >> geneSets, boolean normalizeScores, boolean permutePhenotype) |
|  | |
|  | GeneSetEnrichment (MatrixWithHeaders data, Collection< String > group1, Collection< String > group2, Map< String, Collection< String >> geneSets) |
|  | |
|  | GeneSetEnrichment (MatrixWithHeaders patternData, MatrixWithHeaders geneData, Map< String, Collection< String >> geneSets, boolean normalizeScores, boolean permutePhenotype) |
|  | |
| MatrixWithHeaders | getRankedList () |
|  | |
| MatrixWithHeaders | getKSFDR () |
|  | |
| MatrixWithHeaders | getMaxMeanFDR () |
|  | |
| MatrixWithHeaders | getMaxMeanEnrichments () |
|  | |
| MatrixWithHeaders | getKSEnrichments () |
|  | |
| MatrixWithHeaders | getNormalizedKSEnrichments () |
|  | |
| MatrixWithHeaders | getNormalizedMaxMeanEnrichments () |
|  | |

## Constructor & Destructor Documentation

|  |  |  |  |
| --- | --- | --- | --- |
| broad.projection.gsa.GeneSetEnrichment.GeneSetEnrichment | ( | MatrixWithHeaders | *data*, |
|  |  | Collection< String > | *group1*, |
|  |  | Collection< String > | *group2*, |
|  |  | Map< String, Collection< String >> | *geneSets*, |
|  |  | boolean | *normalizeScores*, |
|  |  | boolean | *permutePhenotype* |
|  | ) |  |  |

|  |  |  |  |
| --- | --- | --- | --- |
| broad.projection.gsa.GeneSetEnrichment.GeneSetEnrichment | ( | MatrixWithHeaders | *data*, |
|  |  | Collection< String > | *group1*, |
|  |  | Collection< String > | *group2*, |
|  |  | Map< String, Collection< String >> | *geneSets* |
|  | ) |  |  |

|  |  |  |  |
| --- | --- | --- | --- |
| broad.projection.gsa.GeneSetEnrichment.GeneSetEnrichment | ( | MatrixWithHeaders | *patternData*, |
|  |  | MatrixWithHeaders | *geneData*, |
|  |  | Map< String, Collection< String >> | *geneSets*, |
|  |  | boolean | *normalizeScores*, |
|  |  | boolean | *permutePhenotype* |
|  | ) |  |  |

## Member Function Documentation

|  |  |  |  |  |
| --- | --- | --- | --- | --- |
| MatrixWithHeaders broad.projection.gsa.GeneSetEnrichment.getKSEnrichments | ( |  | ) |  |

|  |  |  |  |  |
| --- | --- | --- | --- | --- |
| MatrixWithHeaders broad.projection.gsa.GeneSetEnrichment.getKSFDR | ( |  | ) |  |

|  |  |  |  |  |
| --- | --- | --- | --- | --- |
| MatrixWithHeaders broad.projection.gsa.GeneSetEnrichment.getMaxMeanEnrichments | ( |  | ) |  |

|  |  |  |  |  |
| --- | --- | --- | --- | --- |
| MatrixWithHeaders broad.projection.gsa.GeneSetEnrichment.getMaxMeanFDR | ( |  | ) |  |

|  |  |  |  |  |
| --- | --- | --- | --- | --- |
| MatrixWithHeaders broad.projection.gsa.GeneSetEnrichment.getNormalizedKSEnrichments | ( |  | ) |  |

|  |  |  |  |  |
| --- | --- | --- | --- | --- |
| MatrixWithHeaders broad.projection.gsa.GeneSetEnrichment.getNormalizedMaxMeanEnrichments | ( |  | ) |  |

|  |  |  |  |  |
| --- | --- | --- | --- | --- |
| MatrixWithHeaders broad.projection.gsa.GeneSetEnrichment.getRankedList | ( |  | ) |  |

---

The documentation for this class was generated from the following file:

- java/broad/projection/gsa/GeneSetEnrichment.java


---

Generated on Fri Aug 15 2014 15:11:05 for ESAT by  

 1.8.7
